# Tumor-Infiltrating Immune Cells and PD-L1 as Prognostic Biomarkers in Primary Esophageal Small Cell Carcinoma

**DOI:** 10.1155/2020/8884683

**Published:** 2020-12-29

**Authors:** Xiao Wu, Xiurong Ke, Yangpeng Ni, Liping Kuang, Fan Zhang, Yusheng Lin, Wan Lin, Xiao Xiong, Haihua Huang, Xianjie Lin, Hao Zhang

**Affiliations:** ^1^Cancer Research Center, Shantou University Medical College, Shantou, Guangdong, China; ^2^Department of Surgery, Laboratory for Translational Surgical Oncology, University of Groningen, University Medical Center Groningen, Groningen, Netherlands; ^3^Department of Pathology, Jieyang People's Hospital (Jieyang Affiliated Hospital, Sun Yat-Sen University), Jieyang, Guangdong, China; ^4^Department of Pathology, Shantou Central Hospital, Affiliated Shantou Hospital of Sun Yat-Sen University, Shantou, Guangdong, China; ^5^Guangdong Provincial Key Laboratory for Breast Cancer Diagnosis and Treatment, Cancer Hospital of Shantou University Medical College, Shantou, Guangdong, China; ^6^Department of Hematology, University of Groningen, University Medical Center Groningen, Groningen, Netherlands; ^7^Department of General Surgery, The First Affiliated Hospital of Jinan University and Institute of Precision Cancer Medicine and Pathology, Jinan University Medical College, Guangzhou, Guangdong, China; ^8^Department of Pathology, The Second Affiliated Hospital of Shantou University Medical College, Shantou, Guangdong, China; ^9^Research Center of Translational Medicine, The Second Affiliated Hospital of Shantou University Medical College, Shantou, Guangdong, China

## Abstract

Primary esophageal small cell carcinoma (PESCC) is a weakly prevalent but lethal malignancy with early metastasis and a poor prognosis. Currently, neither effective prognostic indicators nor curative therapies are available for PESCC. Immunotherapy has now evolved into one of the most promising therapies for cancer patients. Tumor-infiltrating immune cells which are integral to the tumor immune microenvironment (TIME) are recognized as highly important for prognosis prediction, while the responsiveness to immune checkpoint blockade may be subject to the features of TIME. In this study, we aim to identify the TIME and provide indication for the applicability of immune checkpoint therapy in PESCC. We found that PD-L1 expression was detected in 33.33% (27/81) of all the patients, mostly exhibiting a stroma-only pattern and that it was positively associated with tumor-infiltrating immune cells (CD4^+^, CD8^+^, and CD163^+^). In 74.07% of PD-L1-positive specimens, PD-L1^+^CD163^+^ cells were colocalized more with CD4^+^ than CD8^+^ T cells. 83.95% (68/81) of all the specimens were infiltrated with more CD4^+^ than CD8^+^ T cells. Further analysis showed FoxP3^+^ Tregs constituted 13-27% of the total CD4^+^ T cell population. The Kaplan-–Meier analysis indicated several factors that contribute to poor survival, including negative PD-L1 expression, rich CD4 expression, rich FoxP3 expression, a low CD8/CD4 ratio, and a high FoxP3/CD8 ratio. A nomogram model was constructed and showed good performance for survival prediction. These results highlight that a suppressive TIME contributes to poor survival of patients with PESCC. TIME analyses might be a promising approach to evaluate the possibility and effect of immune checkpoint-based immunotherapeutics in PESCC patients.

## 1. Introduction

Primary esophageal small cell carcinoma (PESCC) is a rare but fast-growing tumor that exhibits a neuroendocrine phenotype and accounts for 0.5-2.8% of all esophageal malignancies [1]. PESCC is featured with early dissemination and poor clinical outcomes [2, 3]. Current treatment options for PESCC are those universal for common malignancies, including surgery, chemotherapy, radiotherapy, and concurrent chemoradiotherapy based on the tumor-node-metastasis (TNM) staging system [4]. However, the prognosis can vary significantly among patients with the same TNM stage, and there remains no targeted or curative therapy due to a lack of knowledge about the mechanisms underlying the cause and progression of PESCC.

Tumor immune microenvironment (TIME) is an important regulator of the antitumor immune response, since immune factors in the TIME have significant impacts on cancer patient prognosis [3, 5–7]. Antibodies against the programmed cell death protein 1 (PD-1) receptor and its ligand PD-L1, among other immune checkpoint inhibitors, have delivered unprecedented clinical benefit in various human malignancies, including melanoma, non-small-cell lung cancer (NSCLC), head and neck squamous cell carcinoma, and bladder cancer [8–11]. Clinical trials have demonstrated the effectiveness of pembrolizumab and nivolumab (PD-1 inhibitors) in small cell lung cancer (SCLC) patients [12–14]. Given that the clinicopathologic features of PESCC are similar to those of SCLC, PD-L1/PD-1-based immunotherapy might be feasible to treat PESCC. Currently, the expression of PD-L1, as detected by immunohistochemical staining, is an important indicator for the use of PD-L1/PD-1 inhibitors in patients with lung cancer and melanoma [15]. However, growing evidence indicates that PD-L1 as a single biomarker is not precise enough to predict the response to PD-L1/PD-1 inhibition. Other factors, such as tumor-infiltrating lymphocyte (TIL) subsets, also need to be taken into consideration [16–18]. Most studies of PESCC are case reports focusing on individuals, and no systematic investigation of the PD-L1 expression pattern and immune cells within the TIME of PESCC has been reported.

Here, we performed a holistic assessment of the expression frequency of PD-L1 in tumor and tumor-infiltrating immune cells (CD4^+^, CD8^+^, and FoxP3^+^ T cells and CD163^+^ macrophages) to get a comprehensive landscape of the TIME in PESCC and provide practicable markers for patient enrollment in future clinical trials. Furthermore, we constructed a prognostic nomogram to predict the survival of PESCC patients, aiming to better classify patients for checkpoint inhibitor immunotherapy.

## 2. Materials and Methods

### 2.1. Patient Selection and Specimens

A total of 81 patients with PESCC who received surgical resection or biopsy in 4 hospitals between 2010 and 2017 were recruited for this research. All recruited patients met the following criteria: (1) tumors which were primary, and recurrences were excluded; (2) pathologically confirmed small cell carcinoma according to the 2017 WHO classification of neuroendocrine carcinomas (NECs); (3) clinical staging of tumors in terms of TNM classification system of the American Joint Committee on Cancer (AJCC, 8th edition); and (4) complete clinicopathologic and follow-up data. This study was approved by the Institution Ethics Committee and Institutional Review Board of all participating institutions, and all procedures were conducted in accordance with the 1975 Declaration of Helsinki. Written informed consent was obtained from all participants.

### 2.2. PESCC Histologic Diagnoses

Histological diagnoses were given following an extensive review of all specimens by 2 independent pathologists (Wu and Zhang). Morphological features were evaluated on hematoxylin and eosin (H&E) slides: tumor cells are small, usually less than the sizes of 3 small lymphocytes, and are shaped with round, ovoid, or spindled nuclei and scant cytoplasm. Nuclear chromatin is finely granular, and nucleoli are invisible or inconspicuous. Cell borders are hardly seen, and nuclear mitosis is commonplace (at least 20 mitoses per 2 mm^2^). Densely packed small tumor cells commonly display a sheet-like diffuse architecture. Architectural patterns, such as nesting, trabeculae, peripheral palisading, and rosette formation (as seen in other neuroendocrine tumors), are comparatively rare in PESCC. Comedo necrosis, extensive necrosis, brisk apoptotic activity, and the Azzopardi effect may all be present (Figure S1A-C). Standard immunohistochemical markers were also applied to the diagnosis of each patient: neuroendocrine markers (chromogranin A, synaptophysin, CD56, or neuron-specific enolase), Ki67, and cytokeratin-Pan (AE1/AE3). Samples were diagnosed as NEC if positive for one or more neuroendocrine markers and have more than 20% of the Ki67 labeling index (Figure S2). Patients with NEC in other organs were excluded.

### 2.3. Distinguishing Tumor and Stroma

Stroma that sustains cancer cells mainly consists of the basement membrane, fibroblasts, extracellular matrix, immune cells, and vasculature [19]. As mentioned above, the tumor niche was identified according to the specific morphological features of malignant cells; the stroma area without cancer cells could be clearly distinguished. As we and others observed, PESCC shows mild or no immune cell infiltration in the tumor niche. Notably, lymphocytes and plasma cells, among other immune cells in the stroma, tend to be lined or goblet-shaped, but lymphoid follicles are rarely formed at the tumor boundary, and capillary infiltration is more likely to be seen (Figure S1).

### 2.4. Evaluation of Tumor-Infiltrating Immune Cells by H&E Staining

A full-section H&E slide was screened for tumor-infiltrating immune cells (including lymphocytes, macrophages, and plasma cells) and defined as poor (no immune cells or a mild and patchy immune cell pattern at the tumor margin) or rich (prominent band-like or florid cup-like immune cells at the invasive edges) (Figure S1D–E) [20] by 2 pathologists (Wu and Zhang) who were blinded to the clinical outcome under a multiheaded microscope. Then, populations of different immune cells were evaluated using immunohistochemistry.

### 2.5. Immunohistochemistry Staining

IHC staining was performed using anti-PD-L1 antibody (1 : 500) (clone 28-8, Abcam, Cambridge, UK) [21–24], anti-CD4 antibody (1 : 500) (clone EPR6855, Abcam, Cambridge, UK), anti-CD8 antibody (1 : 200) (rabbit polyclonal anti-CD8, Abcam, Cambridge, UK), anti-CD163 antibody (1 : 500) (clone EPR19518, Abcam, Cambridge, UK), and anti-FoxP3 antibody (1 : 100) (clone 236A/E7, Abcam, Cambridge, UK) as the primary antibodies.

Briefly, serial 4 *μ*m tissue sections from paraffin blocks were prepared, and formalin-fixed and paraffin-embedded (FFPE) tissue sections were deparaffinized and dehydrated in xylene and graded ethanol solutions. Antigen retrieval was conducted in Tris-EDTA buffer (pH 9.0). A peroxidase-labeled secondary antibody (EnVision/HRP system, DAKO, Carpinteria, CA) was used to visualize antigen, and the DAKO Catalyzed Signal Amplification System for rabbit/mouse antibodies was used for staining and detection (DAKO, Carpinteria, CA). Normal IgG was used as the negative control and tonsil tissue as positive.

### 2.6. Evaluation of PD-L1 Expression and Tumor-Infiltrating Immune Cells by Immunohistochemistry Staining

IHC staining was double-blinded examined by 2 pathologists (Wu and Zhang). PD-L1 positive samples were identified using combined positive score (CPS)—the ratio of all membranous expression on tumor cells and immune cells in stroma (e.g., lymphocytes and macrophages) to the total number of viable tumor cells. Sample with a CPS ≥ 1% will be considered as PD-L1 positive [13, 25].

Tumor-infiltrating CD4^+^, CD8^+^, FoxP3^+^, and CD163^+^ cells were counted in 5 high-power monitor fields (HPFs) in the area of the highest immune cell density (hot spots) using a 40× objective lens, and the values were averaged. For statistical analysis, patients were divided into CD4-, CD8-, FoxP3-, or CD163-poor and rich groups using the mean number of tumor-infiltrating immune cell subsets among all patients as the cutoff point. In addition, the FoxP3/CD8 and CD8/CD4 ratios were computed for each specimen, and the averages were compared.

### 2.7. Immunofluorescence Staining

FFPE tissue sections were deparaffinized and dehydrated in xylene and graded ethanol solutions in preparation for PD-L1, CD4, CD8, FoxP3, and CD163 multicolor immunofluorescence (multi-IF) staining. All slides were placed in citrate buffer (pH 6.0) for heat-induced epitope retrieval and incubated in 3% hydrogen peroxide solution and blocking buffer for blocking endogenous tissue peroxidases. Then, the slides were incubated with anti-PD-L1 (clone 28-8, Abcam, Cambridge, UK), anti-CD4 (clone EPR6855, Abcam, Cambridge, UK), anti-CD8 (rabbit polyclonal anti-CD8, Abcam, Cambridge, UK), anti-FoxP3 (clone 236A/E7, Abcam, Cambridge, UK), and anti-CD163 (clone EPR19518, Abcam, Cambridge, UK) primary antibodies, and HRP-conjugated streptavidin served as the secondary antibody. For IF, slides were visualized with Alexa Fluor 488, Alexa Fluor 594, and Alexa Fluor 647 Tyramide SuperBoost kits (Invitrogen, Carlsbad, CA), and visualization of nuclei was achieved by using ProLong Diamond Antifade Reagent with 4′,6-diamidino-2-phenylindole (DAPI; Invitrogen, Carlsbad, CA). Both primary and secondary antibodies were stripped with citrate buffer (pH 6.0) in the microwave. Tumor tonsil tissues were used as positive control samples (Figure S3).

Analysis of multi-IF staining was performed using the PerkinElmer Vectra (v1.0; PerkinElmer) platform. Single-stained tissue for each reagent was scanned to build spectral libraries to unmix the multispectral images by using Inform Advanced Image Analysis software (Inform v2.1.0; PerkinElmer). Tissue segmentation, cell segmentation, and phenotyping were performed with Inform Advanced Image Analysis software.

### 2.8. Statistical Analyses

Statistical analyses were conducted in IBM SPSS statistics (version 25) software. The association between PD-L1 protein expression and clinicopathologic characteristics was analyzed using the chi-square test or Fisher's exact test. The Wilcoxon test was applied to compare continuous variables of the two groups. The Kaplan–Meier models were used for survival analysis. To detect independent predictive factors for aggressive features of PESCC, a Cox proportional hazards regression model was applied. Covariates with *p* values < 0.01 in the univariate analysis were included in the multivariate analysis. Odds ratios (ORs) and their 95% confidence intervals (CIs) were estimated for each factor. *p* < 0.05 (two-tailed) was considered statistically significant.

### 2.9. Development of the Prognostic Nomogram

A nomogram that demonstrated prognostic significance of different risk factors in a single figure was established using the rms package in R (version 3.4.2). Included factors for creating the prognostic nomogram were selected in accordance with the Cox proportional hazards regression model using backward stepwise selection.

## 3. Results

### 3.1. Patient Demographics and Clinicopathological Characteristics

The diagnosis of PESCC in this study was confirmed by hematoxylin and eosin (H&E) staining and IHC staining for neuroendocrine markers, epithelial markers, or TTF1 and Ki67 (Figures S1 and S2). Most patients (79 of 81) had pure PESCC that did not have significant components of invasive adenocarcinoma or squamous cell carcinoma, whereas 2 patients contained a minor component (less than 20%) of invasive squamous cell carcinoma and 30.86% (25/81) of patients presented extensive necrosis. The basic characteristics of these patients are summarized in Table S1. The cohort of patients averaged was 63 years old (from 40 to 78 years). Most tumors (74 of 81) were located between the middle and lower esophagus, with only 7 tumors located in the upper esophagus. Frontline treatment varied with histology and stage. In this study, 45 patients received surgical treatment, among whom 14 underwent postoperative chemotherapy after surgery and 10 received postoperative radiochemotherapy after surgery because of a positive margin. Among the 36 patients who already had distant metastasis or other underlying health conditions that indicated their unsuitability for surgery, 27 received chemotherapy only, while 9 received radiochemotherapy only. No patient received immunotherapy (Table S2).

### 3.2. The PD-L1 Protein Is Expressed Mainly in the Adjacent Stroma and Is Associated with Overall Survival in PESCC

**Figure 1:**
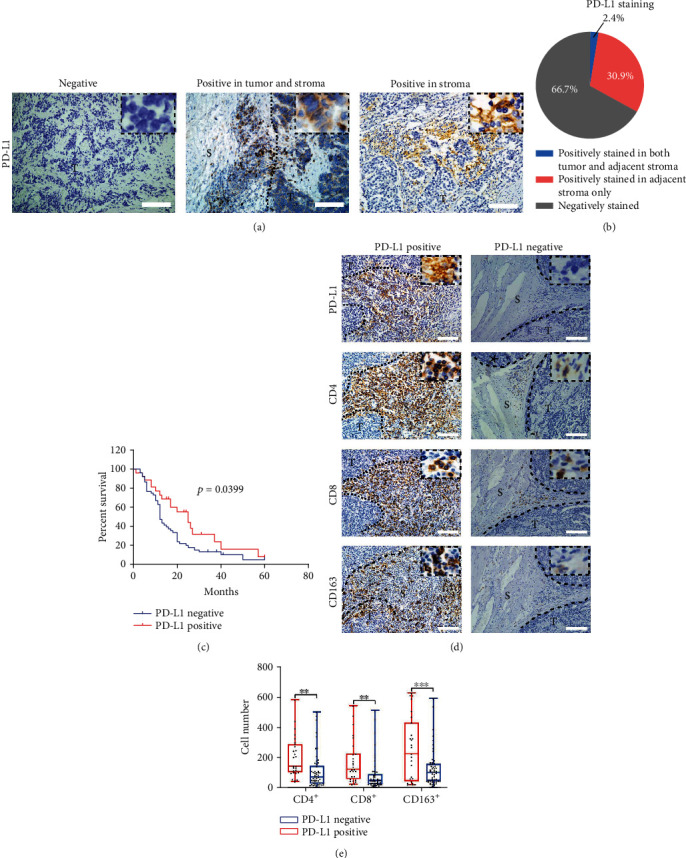
The expression of PD-L1 in the stroma correlates with tumor-infiltrating immune cells in PESCC. (a) Representative immunohistochemistry (IHC) images showing different expression patterns of PD-L1 in PESCC tissues. Scale bar, 100 *μ*m. S: stroma; T: tumor. (b) Distribution of different expression patterns of PD-L1 are plotted in the pie chart. (c) The Kaplan–Meier survival analysis of overall survival (OS) in a cohort of 77 PESCC patients according to positive (red line, *n* = 26) and negative (blue line, *n* = 51) PD-L1 expression. (d) Representative IHC images showing PD-L1, CD4, CD8, and CD163 expression in the stroma using consecutive sections of PD-L1-positive and PD-L1-negative specimens. Scale bar, 100 *μ*m. S: stroma; T: tumor. (e) The presence of PD-L1 is positively associated with the number of CD4^+^ TILs, CD8^+^ TILs, and CD163^+^ TAMs (^∗∗^*p* < 0.01 and ^∗∗∗^*p* < 0.001 by the Wilcoxon test).

PD-L1 protein expression was examined by IHC. Three patterns of PD-L1 expression within PESCC tumor tissue were found in these specimens: PD-L1 negative, PD-L1 positive with staining in the stroma only, and PD-L1 positive with staining in both the tumor and stroma (Figure 1(a)). Unexpectedly, unlike findings observed in oral tongue squamous cell carcinoma (OTSCC) and NSCLC [26, 27], PD-L1 was not expressed exclusively in tumor cells. Of the 81 tumors, 66.67% (54/81) showed negative for PD-L1, and 33.33% (27/81) showed positive for PD-L1. Among the PD-L1-positive tumors, 25 were detected with PD-L1 expression in the stroma only, and 2 were detected with PD-L1 protein expression in both tumor cells and inflammatory cells of the adjacent stroma, with relatively weak staining in tumor cells (Figures 1(a) and 1(b), Table S3). In coincidence with the observation in OTSCC [26], strong PD-L1 staining intensity was observed in areas of tissue necrosis (Figure S4). The concordance of PD-L1 status between the 2 pathologists who analyzed the specimens (Wu and Zhang) was 97%. When patients were categorized into a PD-L1-positive group and a PD-L1-negative group for the correlation between PD-L1 expression and clinicopathological characteristics, the results showed that PD-L1 expression was not correlated with sex, age, tumor location, tumor depth, lymphatic metastasis, distant metastases, necrosis, or tumor stage (Table 1). Further, the Kaplan–Meier analysis demonstrated that PD-L1-positive patients experienced prolonged overall survival (*p* < 0.05, log-rank test) (Figure 1(c)). Multivariate Cox regression analysis showed that PD-L1 expression remained significantly correlated with favorable overall survival in PESCC after adjusting for other clinical factors (HR = 0.48, 95% CI: 0.27–0.86; *p* = 0.014; Table 2). This result indicated that PD-L1 expression was an independent prognostic predictor of PESCC.

### 3.3. PD-L1 Expression in the Stroma Positively Correlates with Tumor-Infiltrating Immune Cells

Since PD-L1 was enriched in the adjacent stroma rather than in the tumor nest, to systematically analyze the TIME in PESCCs, where PD-L1 expression is enriched, we performed H&E staining and IHC analysis of CD4^+^ and CD8^+^ TILs and CD163^+^ tumor-associated macrophages (TAMs) in all 81 patients. According to morphological identification and counting on H&E slides, 44.44% (36/81) comprised a tumor-infiltrating immune cell-rich group, which showed a positive correlation with PD-L1 expression (Table 1, Table S4). Furthermore, CD4-, CD8-, and CD163-expressing cells were counted, and patients were identified as having rich or poor expression by using the mean number of positive cells as the cutoff point. The mean number of tumor-infiltrating CD4^+^, CD8^+^, and CD163^+^ cells was 142 (range 2-580), 113 (range 1-540), and 170 (range 3-624) per high-power field, respectively. We found a positive correlation between CD4^+^, CD8^+^, and CD163^+^ cells versus PD-L1 expression in the stroma (chi-squared test, *p* = 0.0023, *p* = 0.002, and *p* < 0.001, respectively) (Figure 1(d), Table 1, Table S4). Using the Wilcoxon test, we obtained similar results showing that PD-L1 expression was positively correlated to CD4^+^ TILs, CD8^+^ TILs, and CD163^+^ TAMs (*p* < 0.05, *p* < 0.05, and *p* < 0.001, respectively) (Figure 1(e)).

### 3.4. A Higher Frequency of CD4^+^ TILs Colocalized with PD-L1^+^CD163^+^ TAMs Than with CD8^+^ TILs

**Figure 2:**
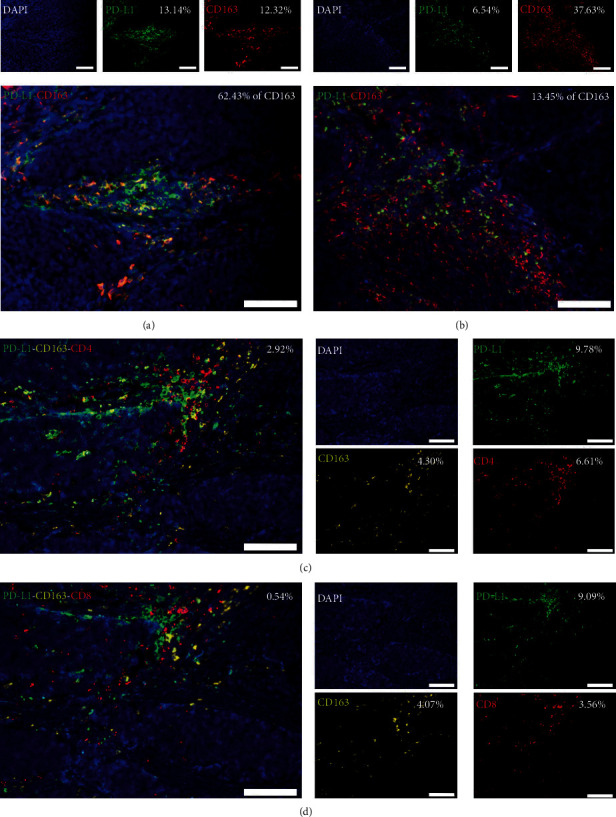
CD4^+^ TILs colocalize with PD-L1^+^CD163^+^ TAMs. Representative multicolor immunofluorescence (multi-IF) images showing (a) high and (b) low colocalization of PD-L1^+^ cells with CD163^+^ TAMs. Scale bar, 100 *μ*m. Representative multi-IF images showing costaining of (c) CD4^+^PD-L1^+^CD163^+^ TILs and (d) CD8^+^PD-L1^+^CD163^+^ TILs in serial sections of the same specimen. Cells were counterstained with DAPI (blue, nucleus). Scale bar, 100 *μ*m.

As described above, immune infiltration frequency was associated with PD-L1 expression within the stroma. We further explored the relationship between PD-L1^+^CD163^+^ TAMs and TILs on the basis of a previous report that demonstrated the PD-L1^+^ TAM is an ideal indicator for PD-1/PD-L1 blockade treatment [28]. We used multi-IF analyses to confirm that PD-L1^+^CD163^+^ TAMs were located mostly within the tumor stroma (Figures 2(a) and 2(b)), and in patients with necrosis, PD-L1^+^CD163^+^ TAMs were observed within necrotic tissue (Figure S4). Furthermore, in all 27 PD-L1-positive specimens, 20 of them had a higher frequency of CD4^+^ TIL infiltration. And in these 20 samples, PD-L1^+^CD163^+^ TAMs colocalized more with CD4^+^ TILs than CD8^+^ TILs (Figures 2(c) and 2(d) and Figure S5).

### 3.5. CD4^+^ TILs Are the Most Clinical Outcome-Related Components among Inflammatory Cells in the Stroma and Positively Correlate with PD-L1

**Figure 3:**
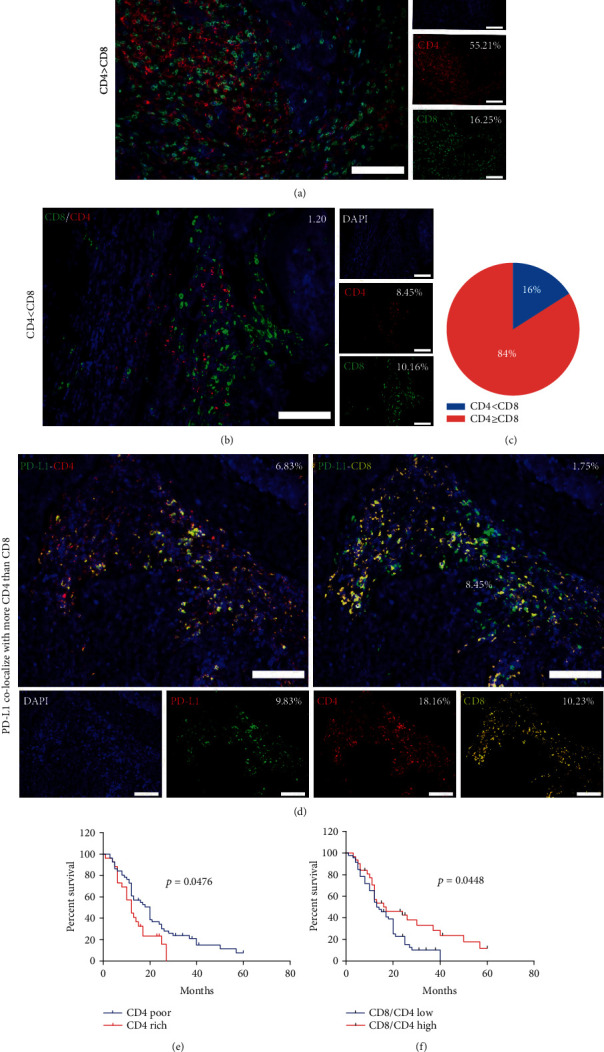
CD4^+^ TILs colocalize with PD-L1^+^ cells and contribute to poor survival. (a) Representative multi-IF images showing a sample with more CD4^+^ TILs than CD8^+^ TILs in the stroma. Scale bar, 100 *μ*m. (b) Representative multi-IF images showing a sample with more CD8^+^ TILs than CD4^+^ TILs in the stroma. Scale bar, 100 *μ*m. (c) A pie chart was plotted according to IHC staining results showing 84% of patients have more CD4^+^ TILs than CD8^+^ TILs in the stroma. (d) Representative multi-IF images showing a sample with PD-L1^+^ cells colocalizing more with CD4^+^ TILs than with CD8^+^ TILs in the stroma. Scale bar, 100 *μ*m. (e) The Kaplan–Meier survival analysis showing patients with elevated levels of CD4^+^ TILs (red line, *n* = 26) have poor OS, compared to patients with low levels of CD4^+^ TILs (blue line, *n* = 51). (f) The Kaplan–Meier survival analysis showing patients with a low CD8/CD4 ratio (blue line, *n* = 46) have poor OS, compared to patients with a high CD8/CD4 ratio (red line, *n* = 31).

Notably, IHC analysis of CD4^+^ and CD8^+^ T cell proportions revealed that most specimens (68/81) had more CD4^+^ TILs than CD8^+^ TILs (Figure 3(c)), in agreement with the results of multi-IF staining for CD4 and CD8 (Figures 3(a) and 3(b)). In addition, more CD4^+^ TILs colocalized with PD-L1 than CD8^+^ TILs (Figure 3(d)), suggesting that CD4^+^ TILs are more responsible for PD-L1-induced anergic lymphocytes than CD8^+^ TILs and indicating the involvement of CD4^+^ TILs in PD-L1/PD-1 function in PESCC. Furthermore, the Kaplan–Meier analysis of overall survival revealed that patients whose tumors were rich in CD4^+^ TILs experienced shorter survival (Figure 3(e)). When patients were classified by CD8^+^ TILs, tumor-infiltrating immune cells, or CD163^+^ cells (poor or rich), their overall survival showed no significant difference (Figure S6A–C). However, a higher CD8/CD4 T cell ratio was associated with a more desirable clinical outcome (Figure 3(f)).

### 3.6. A Low FoxP3^+^/CD8^+^ T Cell Ratio Correlates with Good Clinical Outcomes in PESCC Patients

**Figure 4:**
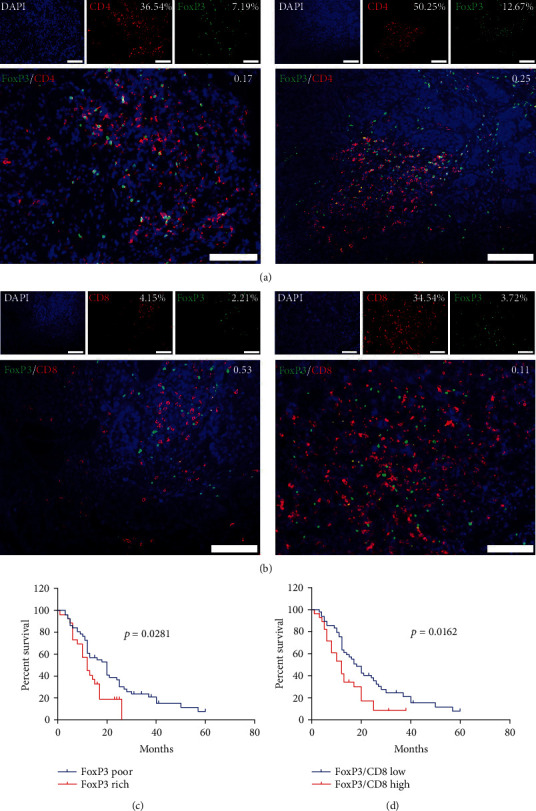
Expression and prognostic value of FoxP3 in PESCC. (a) Representative multi-IF images of two samples showing FoxP3/CD4 ratios in the stroma. Scale bar, 100 *μ*m. (b) Representative multi-IF images showing two samples with high (left) and low (right) FoxP3/CD8 ratios in stroma. Scale bar, 100 *μ*m. (c) Rich FoxP3 (red line, *n* = 26) was associated with significantly shorter OS than poor FoxP3 (blue line, *n* = 51). (d) A high FoxP3/CD8 ratio (red line, *n* = 28) was associated with shorter OS than a low FoxP3/CD8 ratio (blue line, *n* = 49).

Since we identified a predominant contribution of CD4^+^ TILs in this cohort, which was associated with short overall survival, we suspected that CD4^+^FoxP3^+^ regulatory T cells (Tregs) might play an instrumental role in this context. Besides examining all 81 samples with IHC staining for FoxP3 (mean number: 25 per field, range 0-130 per field), we further selected 8 samples on a random basis for multi-IF analysis. As expected, Tregs constituted 13-27% of the total CD4^+^ T cell population under the multi-IF model (Figure 4(a)). The number of Tregs in the tumor, to a large extent, depended on the abundance of CD4^+^ TILs but not CD8^+^ TILs (Figure 4(b), Table S5). The Kaplan–Meier analysis showed that a small number of Tregs and a low FoxP3/CD8 T cell ratio were correlated with prolonged overall survival (Figures 4(c) and 4(d)).

### 3.7. The TIME Is Different between PD-L1-Positive and PD-L1-Negative PESCC

To further analyze the role of PD-L1 in the TIME of PESCC, prognostic analysis was performed. The Kaplan–Meier analysis showed that a small number of Tregs, as well as a low FoxP3/CD8 T cell ratio and a high CD8/CD4 T cell ratio, were correlated with prolonged overall survival in PD-L1-positive PESCC (Figure S7A–C). When PD-L1-positive patients were classified by tumor-infiltrating immune cell enrichment (or CD8^+^ TIL, CD163^+^ TAM, and CD4^+^ TIL enrichment) and by necrosis harbored in tumor tissue, there was no significant difference in overall survival (Figure S7D–H). In PD-L1-negative PESCC, the prolonged overall survival is related to no necrosis in tumor tissue, a small number of Tregs, and fewer CD4^+^ TILs (Figure S8A–C), while the other indicators had no significant correlation with overall survival (Figure S8D–H).

### 3.8. Development of a Nomogram Based on Independent Prognostic Factors Combining Immune Factors

The clinical manifestation and immune effectors of the patients were brought into the equation and subjected to univariate analysis. Among the clinical parameters, the depth of tumor invasion (T stage), the status of lymph node metastasis (N stage), metastasis (M stage), TNM stage, and necrosis stage had significant association with survival (Table 2). The risk factors with *p* values less than 0.1 in the univariate analysis, including T classification (*p* = 0.014), N classification (*p* < 0.001), M classification (*p* < 0.001), stage classification (*p* = 0.001), necrosis stage (*p* = 0.040), tumor-infiltrating immune cells (*p* = 0.073), CD4 (*p* = 0.059), FoxP3 (*p* = 0.036), CD8 (*p* = 0.064), PD-L1 (*p* = 0.050), FoxP3/CD8 (*p* = 0.022), and CD8/CD4 (*p* = 0.055), were selected for a Cox proportional hazards regression model for multivariate analysis. Since the TNM stage contains T, N, and M stages and tumor-infiltrating immune cells contain CD4, CD8, and FoxP3, we entered TNM stage, CD4, and the FoxP3/CD8 ratio [29] into the multivariate analysis, which revealed one tumor characteristic (TNM stage (*p* = 0.003)) and three immune variables (PD-L1 (*p* = 0.014), CD4 (*p* = 0.030), and FoxP3/CD8 (*p* = 0.044)) that were independent indicators for overall survival and were thereby included in the following predictive model (Table 2).

**Figure 5:**
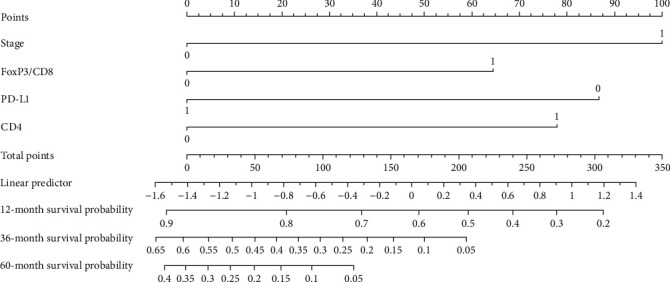
Development of the nomogram for predicting OS in patients with PESCC. To estimate the OS of an individual patient, the value of each factor is acquired on each variable axis, and a straight line is drawn upward to determine the points. The sum of these 4 numbers is located on the total points axis, and then, a line is drawn downward to the survival axes to determine the likelihood of survival.

Although three immune variants showed independent prognostic significance, their intricate interaction within the TIME does not allow any one of these factors to be accurately indicative of survival in PESCC patients. In this sense, it is necessary to identify a comprehensive immunofactor model. A nomogram for overall survival prediction was built using the 4 prognostic factors mentioned above. The nomogram comprises 10 rows, with their representation as follows. The first row (points) represents the point assignment for each variable. For each patient, each variable is assigned a point value in accordance with the clinicopathological features illustrated with a vertical line between the exact variable value and the point line. Subsequently, a total point score (row 6) can be worked out by aggregating all of the assigned points for the 4 variables. The survival likelihood can be obtained by drawing a predictor line (row 7). Then, we draw a line downward to the survival axes to figure out the survival probability (rows 8-10). In this prognostic nomogram, “0” corresponds to stage I-II, FoxP3/CD8 low, CD4^+^ TILs low, and PD-L1 negative, while “1” corresponds to stage III-IV, FoxP3/CD8 high, CD4^+^ TILs high, and PD-L1 positive (Figure 5). Our model was compared with the model that contained only the variable TNM stage in terms of predictive accuracy and goodness of fit. The c-indexes of these two models were 0.710 (95% CI: 0.650-0.771) and 0.635 (95% CI: 0.573-0.697), respectively. The likelihood ratio test for Cox models indicated that based on goodness of fit, the immunofactor model was superior to the model with merely TNM stage (*p* = 0.002).

## 4. Discussion

PESCC is a highly metastatic cancer with a poor outcome. Due to a lack of large-scale clinical studies, opinions on the treatment of PESCC remain controversial, although the importance of surgery has been recognized. Patients with PESCC, even those with early-stage disease, are plagued by high recurrence [30, 31]. To date, the association between the strong aggressiveness and poor prognosis of PESCC and specific biomarker has not been identified in clinical practice. Increasing evidence has indicated that tumor progression is determined by extrinsic immunological factors in the TIME as well as its intrinsic characteristics (for example, TNM stage) [32–34]. Our data showed that in PESCC, PD-L1 is predominantly expressed in the adjacent stroma rather than the tumor nest, which contrasts with non-small-cell solid cancers, such as head and neck squamous cell carcinoma and NSCLC [26, 27]. We found that PD-L1^+^ patients experienced prolonged overall survival, suggesting that PD-L1 expression by the tumor-infiltrating immune cells is correlated with a more beneficial immune response. Further analysis revealed positive correlations between the expression of PD-L1 and tumor-infiltrating immune cells, including TAMs and TILs. PD-L1^+^CD163^+^ cells were colocalized more with CD4^+^ TILs than with CD8^+^ TILs. Besides, we observed a higher frequency of CD4^+^ TILs than CD8^+^ TILs, and the proportion of Tregs was positively correlated to CD4^+^ TILs in these patients. These results elaborate the suppressive TIME status in PESCC patients. In addition to TNM stage, we found that the status of PD-L1, CD4, and the Foxp3/CD8 ratio can predict the outcome of PESCC, and we further constructed a nomogram that included these parameters for survival prediction. Furthermore, the likelihood ratio test showed that this immunofactor model is superior to the model that includes merely TNM stage.

Therapeutic options for PESCC are very limited. The success of immune checkpoint-blocking antibodies in treating melanoma and NSCLC has inspired researchers to introduce this promising immunotherapy into other aggressive solid tumors [13, 14, 35, 36]. Immunohistochemical staining of patient-derived tumor tissues revealed that most patients in our cohort expressed PD-L1 only in the adjacent stroma. Similar to our observation, it has been reported that PD-L1 is negative on tumor cells but positive in the stroma in small cell NECs (including 61 pulmonary and 33 extrapulmonary tumors) [37]. Taken together, these data suggest that small cell carcinomas may display a distinct stromal pattern of PD-L1 compared to non-small-cell carcinomas, in which PD-L1 is expressed mainly on tumor cells. However, investigations using larger sample sizes and with small cell carcinomas from different origins should be conducted to confirm these results. Recent studies verified that the presence of PD-L1 on tumor-infiltrating immune cells has predictive implication for anti-PD-1 therapy [11, 13, 38]. Since patients with NECs have similar morphological and pathological features [39], it is reasonable to hypothesize that PESCC patients with stromal PD-L1 might respond to anti-PD-1/PD-L1 therapy. Therefore, PD-L1 expression in tumor-infiltrating immune cells should also be taken into consideration as a companion diagnostic in clinical trial designs for anti-PD-1/PD-L1 therapy, especially for PESCC.

PD-L1 coexpression with tumor-infiltrating immune cells may act as an adaptive mechanism for immune escape [40–42]. We conducted analyses as per the TIME in the context of PD-L1 expression to evaluate the correlation between PD-L1 and immune cells and to further determine their prognostic values. A study in head and neck squamous cell carcinomas found that Tregs represent only 2 to 15% of CD4^+^ TILs [26]. In the current study, we found that Tregs impressively ranged between 13% and 27% of CD4^+^ TILs in PESCC. Furthermore, PD-L1 was colocalized with CD4^+^ TILs rather than CD8^+^ TILs, in agreement with that in OTSCCs [26]. We showed elevated expression of both CD4^+^ TILs and Tregs, and a low CD8/CD4 ratio correlated with poor overall survival in PESCC. These results suggest that CD4^+^ TILs, especially Tregs, play pivotal roles in the immunosuppressive microenvironment in PESCC. PD-L1 expressed on immune cells of the innate immune system, such as macrophages, can trigger important modulatory effects within the TIME [20, 43]. We found that PD-L1^+^CD163^+^ cells colocalized more with CD4^+^ TILs than with CD8^+^ TILs, suggesting that PD-L1^+^ TAMs may be a regulator of Tregs in PESCC.

The Kaplan–Meier analysis revealed different prognostic values for the overall survival of PD-L1-positive and PD-L1-negative patients. A high CD8^+^/CD4^+^ ratio, a low Foxp3/CD8 ratio, and low Foxp3 expression were associated with prolonged survival in PD-L1-positive patients, while the presence of necrosis, high CD4 expression, and high Foxp3 expression were associated with poor survival in PD-L1-negative patients. Extrinsic (immune-induced) and intrinsic oncogenic activation can regulate PD-L1 expression. We demonstrated that the balance of suppressive and effective TILs is prognostic for the overall survival of PD-L1-positive patients but not for that of PD-L1-negative patients, indicating that the state of the immune system is an important player in the progression of PESCC. Therefore, it is necessary to evaluate the expression of PD-L1 and the proportion of TIL subsets at the same time.

Tumor necrosis is an important hallmark of aggressive cancers. Tumor necrosis induced by hypoxia attracts macrophages to migrate into tumors, which then contribute to angiogenesis and a poor prognosis [44] (Figure S6D). We found that necrosis indicated a poor prognosis in patients with negative PD-L1 expression but not in patients with positive PD-L1 expression. Combining this result with the observation that PD-L1-positive specimens have increased infiltration of CD163^+^ cells in the necrotic areas, we speculate that it is the recruitment of TAMs into the necrotic areas that contributes to the different prognostic roles of necrosis here. However, the expression of CD163 alone is not significantly correlated with overall survival. Further analyses on the functions of these TAMs in small cell carcinomas, particularly in necrotic areas, are still needed.

## 5. Conclusions

In this study, we present a comprehensive picture of the TIME, which is in a suppressive state in PESCC, by examining PD-L1 expression and analyzing its correlation with tumor-infiltrating immune cells. In addition, using prognostic analyses and building a prognostic nomogram based on the independently prognostic immune variables, we provide an alternative model for survival prediction, and we found that the poor survival of PESCC patients was attributed to a suppressive TIME. Future studies remain necessary to compare the predictive accuracy between this model and the TNM staging system in larger PESCC cohorts so as to confirm the predictive value of this model.

## Figures and Tables

**Table 1:** Associations between PD-L1, clinicopathological characteristics, and other immune cells in PESCC.

Characteristic	PD-L1 negative	PD-L1 positive	^a^ *p* value
	No. of patients	No. of patients	
Sex			0.179
Male	38	23	
Female	16	4	
Age			0.465
≤60	22	8	
>60	32	19	
Location			0.681
Upper third	4	3	
Middle and lower thirds	50	24	
^b^T classification			0.811
T1-T2	21	12	
T3-T4	33	15	
^b^N classification			0.220
N0	16	12	
N1	38	15	
^b^M classification			0.742
M0	45	24	
M1	9	3	
^b^Stage			0.472
I-II	20	13	
III-IV	34	14	
Necrosis			0.865
No	37	19	
Yes	17	8	
First treatment			0.518
Surgery	29	16	
Chemoradiotherapy	5	4	
Chemotherapy	20	7	
^c^TIIs			<0.001
Poor	38	7	
Rich	16	20	
CD4			0.023
Poor	41	13	
Rich	13	14	
CD8			0.002
Poor	45	13	
Rich	9	14	
CD163			<0.001
Poor	44	11	
Rich	10	16	

^a^Fisher's test; ^b^TNM stage: the AJCC (8th edition) was used; ^c^TIIs: tumor-infiltrating immune cells.

**Table 2:** Univariate and multivariate analyses for overall survival in PESCC.

Prognostic factor	Univariate analysis			Multivariate analysis		
	HR	95% CI	*p*	HR	95% CI	*p*
Sex						
Male vs. female	0.682	0.389-1.194	0.180			
Age						
≤60 vs. >60	1.384	0.818-2.342	0.226			
Location						
Upper third vs. middle and lower thirds	1.134	0.486-2.647	0.771			
^a^T classification						
T1-2 vs. T3-4	1.964	1.148-3.359	0.014			
^a^N classification						
N0 vs. N1-2	0.363	0.207-0.637	<0.001			
^a^M classification						
M0 vs. M1	3.933	1.945-7.950	<0.001			
^a^Stage						
I-II vs. III-IV	2.493	1.463-4.248	0.001	2.334	1.323-4.118	0.003
Necrosis						
No vs. yes	1.761	1.027-3.021	0.040			
^b^TIIs						
Poor vs. rich	0.628	0.378-1.044	0.073			
CD4						
Poor vs. rich	1.696	0.981-2.934	0.059	1.935	1.068-3.507	0.030
FoxP3						
Poor vs. rich	1.807	1.038-3.145	0.036			
CD8						
Poor vs. rich	0.568	0.312-1.033	0.064			
CD163						
Poor vs. rich	0.762	0.432-1.345	0.349			
PD-L1						
Negative vs. positive	0.578	0.333-1.000	0.050	0.480	0.267-0.860	0.014
FoxP3/CD8						
Low vs. high	1.842	1.091-3.108	0.022	1.727	1.015-2.938	0.044
CD8/CD4						
Low vs. high	0.590	0.344-1.012	0.055			

^a^TNM stage: the AJCC (8th edition) was used; ^b^TIIs: tumor-infiltrating immune cells.

## Data Availability

The data supporting the conclusions of this article are included in the article.

## Supplementary Materials

Supplementary Materials Figure S1: morphological features of PESCC on H&E staining. (A) Overview of a PESCC tumor showing sheets of small cells, with scant cytoplasm, finely granular nuclear chromatin, and an absence of nucleoli. (B) PESCC with high capillary infiltration and the Azzopardi effect can also be seen. Green arrows indicate the microvasculature. (C) PESCC with extensive necrosis. The green arrow indicates necrosis. (D) Poor tumor-infiltrating immune cells at the margin of the tumor bed. (E) High tumor-infiltrating immune cells at the margin of the tumor nest. Scale bar 100 *μ*m for the left panels and 50 *μ*m for the right panels. S: stroma; T: tumor. Figure S2: a representative PESCC sample stained by IHC. (A) Syn-positive tumor cells. (B) CD56-positive tumor cells. (C) CK-Pan-positive tumor cells. (D) Tumor with more than 20% of Ki67-positive cells. Scale bar 100 *μ*m for the left panels and 50 *μ*m for the right panels. Figure S3: human tonsil tissue serves as positive control for IHC and immunofluorescence. (A and B) PD-L1 expressed in crypt epithelium. (C) FoxP3 expressed in lymphoid tissue. (D) CD163 expressed in crypt epithelium and margin connective tissue macrophage. (E) CD4 expressed in lymphoid tissue. (F) CD8 expressed in lymphoid tissue. Scale bar, 100 *μ*m. Figure S4: PD-L1-positive necrotic tissue accompanied with high CD163^+^ TAMs. IHC images of PD-L1, CD4, CD8, and CD163 in necrotic tissue in consecutive sections of 2 specimens. N: necrosis; T: tumor. Scale bar, 100 *μ*m. Figure S5: PD-L1^+^CD163^+^ cells colocalize with TILs. IHC (A) and multi-IF images (B and C) show PD-L1^+^CD163^+^ cells were colocalized with more CD4^+^ TILs than CD8^+^ TILs in consecutive sections of 1 case. Scale bar, 50 *μ*m. Figure S6: survival curves for PESCC patients according to types of immune cells and necrosis status. OS of patients grouped by the following. (A) TIIs (tumor-infiltrating immune cells) in PESCC. TIIs poor (blue line), *n* = 43; TIIs rich (red line), *n* = 34. (B) CD8^+^ T cells in PESCC. CD8 poor (blue line), *n* = 56; CD8 rich (red line), *n* = 21. (C) CD163^+^ T cells in PESCC. CD163 poor (blue line), *n* = 53; CD163 rich (red line), *n* = 24. (D) Necrosis in PESCC. No necrosis (blue line), *n* = 53; necrosis (red line), *n* = 24. Figure S7: survival curves for PD-L1^+^ PESCC patients according to tumor-infiltrating immune cells and necrosis status. OS of patients grouped by the following. (A) CD8/CD4 ratio in PD-L1^+^ PESCC. CD8/CD4 low (blue line), *n* = 14; CD8/CD4 high (red line), *n* = 12. (B) FoxP3/CD8 in PD-L1^+^ PESCC. FoxP3/CD8 low (blue line), *n* = 16; FoxP3/CD8 high (red line), *n* = 10. (C) FoxP3^+^ T cells in PD-L1^+^ PESCC. FoxP3 poor (blue line), *n* = 12; FoxP3 rich (red line), *n* = 14. (D) TIIs in PD-L1^+^ PESCC. TIIs poor (blue line), *n* = 7; TIIs rich (red line), *n* = 19. (E) Different CD4^+^ T cell statuses in PD-L1^+^ PESCC. CD4 poor (blue line), *n* = 12; CD4 rich (red line), *n* = 14. (F) Different CD8^+^T cell statuses in PD-L1^+^ PESCC. CD8 poor (blue line), *n* = 13; CD8 rich (red line), *n* = 13. (G) CD163^+^ TAMs in PD-L1^+^ PESCC. CD163 poor (blue line), *n* = 11; CD163 rich (red line), *n* = 15. (H) Necrosis in PD-L1^+^ PESCC. No necrosis (blue line), *n* = 19; necrosis (red line), *n* = 7. Figure S8: survival curves for PD-L1^−^ PESCC patients according to tumor-infiltrating immune cells and necrosis. OS of patients grouped by the following. (A) Necrosis in PD-L1^−^ PESCC. No necrosis (blue line), *n* = 34; necrosis (red line), *n* = 17. (B) CD4^+^ T cells in PD-L1^−^ PESCC. CD4 poor (blue line), *n* = 39; CD4 rich (red line), *n* = 12. (C) FoxP3^+^ T cells in PD-L1^−^ PESCC. FoxP3 poor (blue line), *n* = 39; FoxP3 rich (red line), *n* = 12. (D) CD8^+^ T cells in PD-L1^−^ PESCC. CD8 poor (blue line), *n* = 43; CD8 rich (red line), *n* = 8. (E) CD163^+^ TAMs in PD-L1^−^ PESCC. CD163 poor (blue line), *n* = 42; CD163 rich (red line), *n* = 9. (F) TIIs status in PD-L1^−^ PESCC. TIIs poor (blue line), *n* = 36; TIIs rich (red line), *n* = 15. (G) CD8/CD4 ratio in PD-L1^−^PESCC. CD8/CD4 low (blue line), *n* = 32; CD8/CD4 high (red line), *n* = 19. (H) FoxP3/CD8 ratio in PD-L1^−^PESCC. FoxP3/CD8 low (blue line), *n* = 33; FoxP3/CD8 high (red line), *n* = 18.
