# Supplementary material for: Tumor-Infiltrating Immune Cells and PD-L1 as Prognostic Biomarkers in Primary Esophageal Small Cell Carcinoma
Source: J Immunol Res. 2020 Dec 29;2020:8884683. doi: 10.1155/2020/8884683 (PMC7785377; doi:10.1155/2020/8884683)
Supplement: Supplementary Materials — Figure S1: morphological features of PESCC on H&E staining. (A) Overview of a PESCC tumor showing sheets of small cells, with scant cytoplasm, finely granular nuclear chromatin, and an absence of nucleoli. (B) PESCC with high capillary infiltration and the Azzopardi effect can also be seen. Green arrows indicate the microvasculature. (C) PESCC with extensive necrosis. The green arrow indicates necrosis. (D) Poor tumor-infiltrating immune cells at the margin of the tumor bed. (E) High tumor-infiltrating immune cells at the margin of the tumor nest. Scale bar 100 μm for the left panels and 50 μm for the right panels. S: stroma; T: tumor. Figure S2: a representative PESCC sample stained by IHC. (A) Syn-positive tumor cells. (B) CD56-positive tumor cells. (C) CK-Pan-positive tumor cells. (D) Tumor with more than 20% of Ki67-positive cells. Scale bar 100 μm for the left panels and 50 μm for the right panels. Figure S3: human tonsil tissue serves as positive control for IHC and immunofluorescence. (A and B) PD-L1 expressed in crypt epithelium. (C) FoxP3 expressed in lymphoid tissue. (D) CD163 expressed in crypt epithelium and margin connective tissue macrophage. (E) CD4 expressed in lymphoid tissue. (F) CD8 expressed in lymphoid tissue. Scale bar, 100 μm. Figure S4: PD-L1-positive necrotic tissue accompanied with high CD163+ TAMs. IHC images of PD-L1, CD4, CD8, and CD163 in necrotic tissue in consecutive sections of 2 specimens. N: necrosis; T: tumor. Scale bar, 100 μm. Figure S5: PD-L1+CD163+ cells colocalize with TILs. IHC (A) and multi-IF images (B and C) show PD-L1+CD163+ cells were colocalized with more CD4+ TILs than CD8+ TILs in consecutive sections of 1 case. Scale bar, 50 μm. Figure S6: survival curves for PESCC patients according to types of immune cells and necrosis status. OS of patients grouped by the following. (A) TIIs (tumor-infiltrating immune cells) in PESCC. TIIs poor (blue line), n = 43; TIIs rich (red line), n = 34. (B) CD8+ T cells in PESCC. CD8 poor [file 8884683.f1.zip › supplementary table for jir.docx]

**Supplementary table**


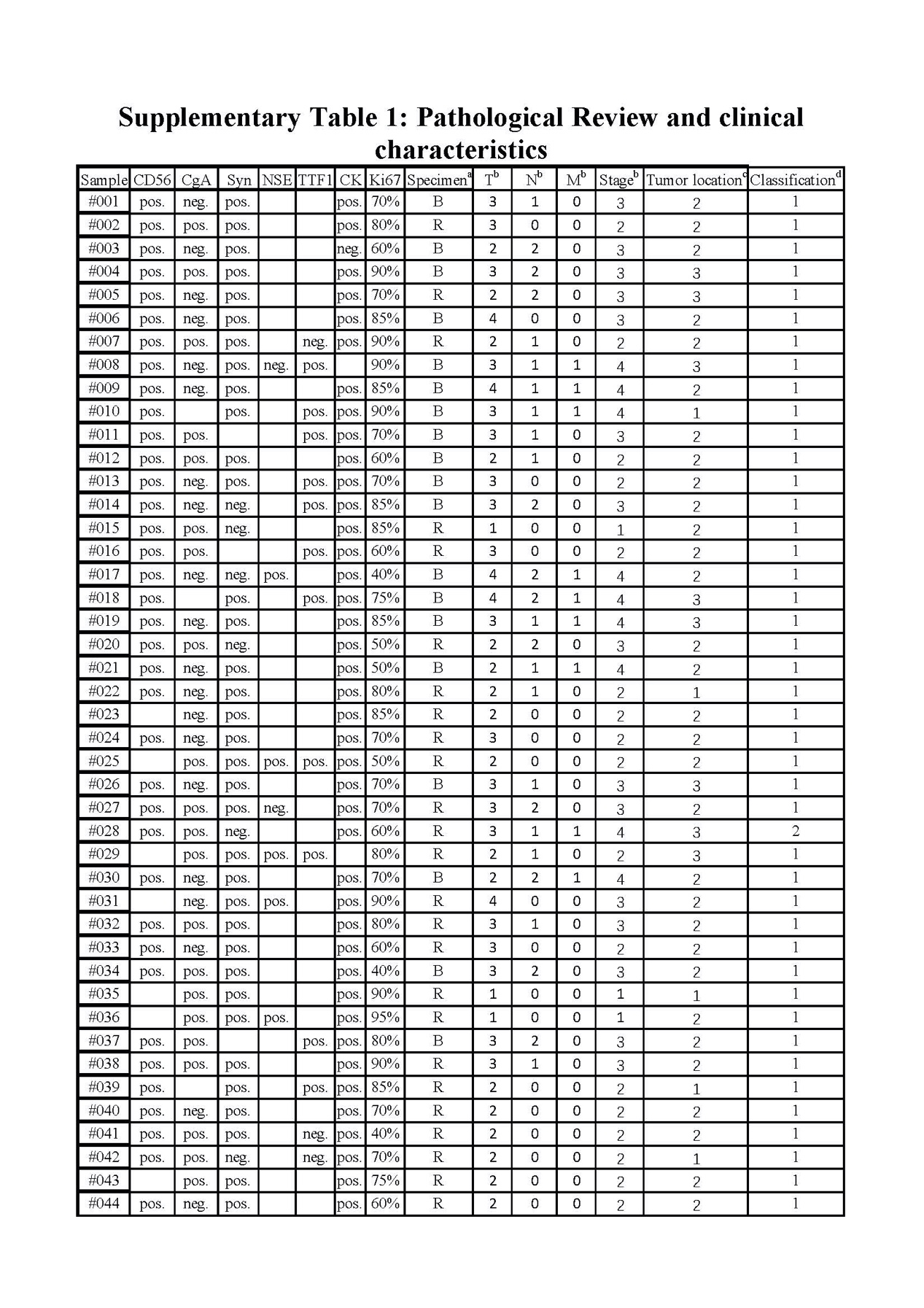


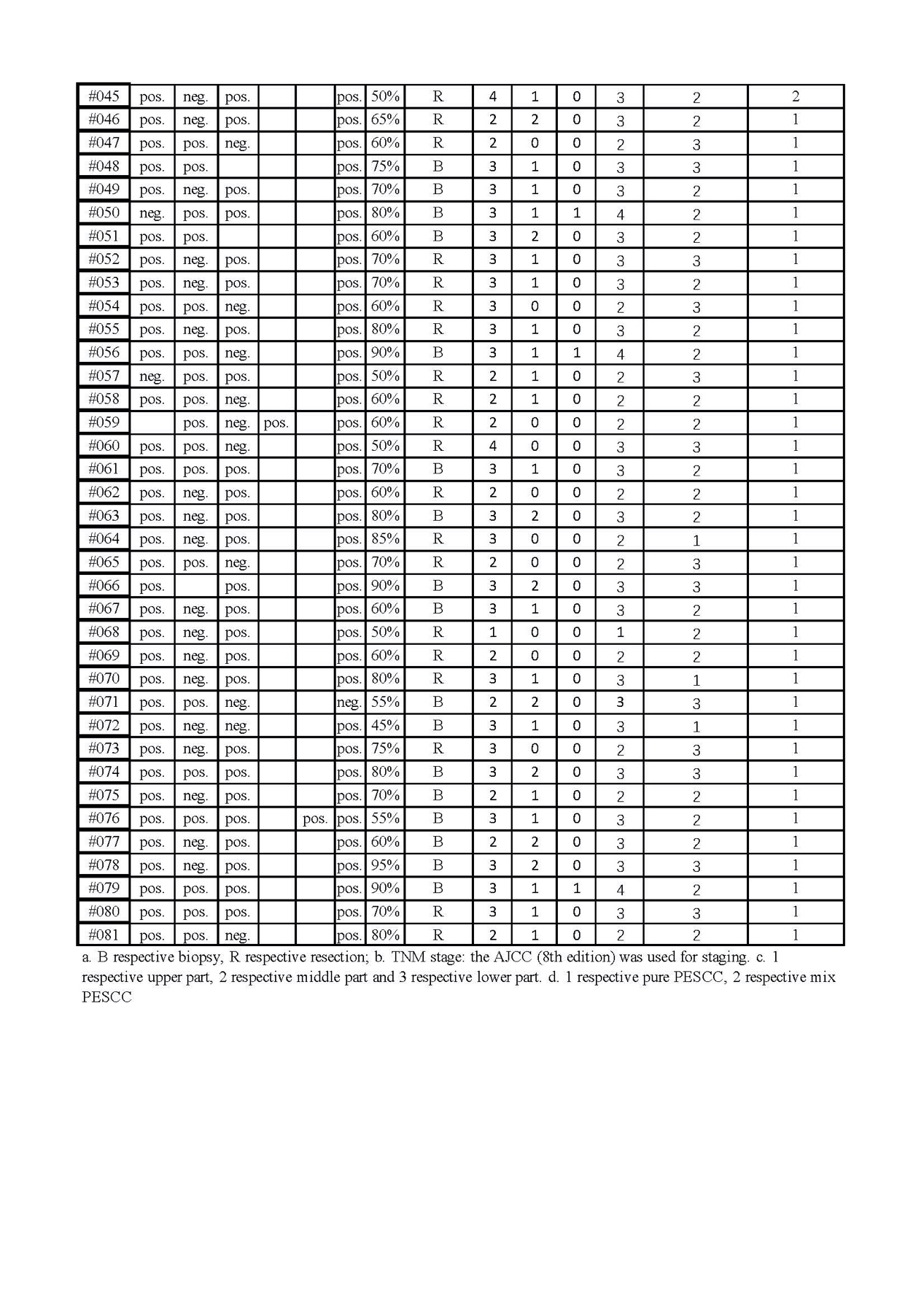


Table S2: Clinicopathological characteristics of PESCC

| Variable | No. of patients（%） | |
| --- | --- | --- |
| Gender |  | |
| Male | 61 (75.3) | |
| Female | 20 (24.7) | |
| Age (mean + SD) | 63.12$\pm8.572$ |  |
| $\leq60$ | 30 (37) | |
| $>60$ | 51 (63) | |
| Location |  | |
| Upper third | 7 (8.6) | |
| Middle and low third | 74 (91.4) | |
| T classification |  | |
| T1-T2 | 33 (40.7) | |
| T3-T4 | 48 (59.3) | |
| N classification |  | |
| N0 | 28 (34.6) | |
| N1 | 53 (65.4) | |
| M classification |  | |
| M0 | 69 (85.2) | |
| M1 | 12 (14.8) | |
| Stage |  | |
| I-II | 33 (40.7) | |
| III-IV | 48 (59.3) | |
| Necrosis |  | |
| No | 56 (69.1) | |
| Yes | 25 (30.9) | |
| First treatment |  | |
| Surgery | 45 (55.6) | |
| Chemo-radiotherapy | 9 (11.1) | |
| Chemotherapy | 27 (33.3) | |

Table S3: PD-L1 staining in the tumor microenvironment

| **Variable** | **No. of patients (%)** |
| --- | --- |
| Positively stained in tumor only | 0 (0) |
| Positively stained in both tumor and adjacent stroma | 2 (2.4) |
| Positively stained in adjacent stroma only | 25 (30.9) |
| Negatively stained | 54 (66.7) |

Table S4: Immune cell expression in PESCC

| Variable | No. of patients（%） |
| --- | --- |
| Tumor-infiltrating immune cells |  |
| Poor | 45 (55.6) |
| Rich | 36 (44.4) |
| CD4 |  |
| Poor | 54 (66.7) |
| Rich | 27 (33.3) |
| CD8 |  |
| Poor | 60 (71.6) |
| Rich | 23 (28.4) |
| FoxP3 |  |
| Poor | 54 (66.7) |
| Rich | 27 (33.3) |
| CD163 |  |
| Poor | 55 (67.9) |
| Rich | 26 (32.1) |
| PD-L1 |  |
| Negative | 54 (66.7) |
| Positive | 27 (33.3) |
| FoxP3/CD8 |  |
| Low | 51 (63) |
| High | 30 (37) |
| CD8/CD4 |  |
| Low | 48 (59.3) |
| High | 33 (40.7) |

Table S5: Relation between CD4 and FoxP3

| Characteristics | CD4 poor | CD4 rich | ^a^*P*-value |
| --- | --- | --- | --- |
|  | No. of patients | No. of patients |  |
| FoxP3 |  |  | <0.001 |
| Poor | 51 | 3 |  |
| Rich | 3 | 24 |  |

a. Fisher’s test.
